# Does your job really matter? Job-specific cancer incidence among a cohort coal mine workers in Queensland, Australia

**DOI:** 10.1007/s00420-025-02188-x

**Published:** 2026-01-07

**Authors:** Deborah Catherine Glass, Stella May Gwini, Anthony Del Monaco, Lin Fritschi, Michael J. Abramson, Malcolm Ross Sim, Karen Walker-Bone

**Affiliations:** 1 Faculty of Medicine Nursing and Health Sciences, Monash Centre for Occupational and Environmental Epidemiology, Monash University, 553 St Kilda Road, Melbourne, VIC 3004 Australia; 2 Division of Health Sciences, School of Public Health, Curtin University, Perth, WA Australia

**Keywords:** Coal mine workers, Cancer incidence, Production workers, Smoking, Retrospective cohort.

## Abstract

**Purpose:**

Globally, coal production exposes millions of workers to coal mine dust. This study aimed to measure overall and site specific cancer incidence among coal mine workers (CMW) who performed different types of work.

**Methods:**

A retrospective cohort of CMW in Queensland, Australia, was assembled using health assessment records from 1992. CMW were grouped by job type and then into Work Categories and linked to the Australian Cancer Database up to 2016. Standardized cancer incidence ratios (SIR) and relative cancer incidence ratios (aRIR) within the cohort, adjusted for age, era, and smoking status, were calculated for men and women with Poisson regression.

**Results:**

There were 5,568 cancers diagnosed among 146,553 men and 396 in 19,927 women. The overall cancer risk was comparable to that of the general population for most Work Categories. The risk of lung cancer was higher for male Production (SIR 123, 95% Confidence Interval [CI] 107–142; adjusted Relative incidence Ratio (aRIR) (adjusted for era, age and smoking status) 1.23, 95%CI 0.99-1.52) and Construction workers (SIR 189, 95%CI 31–272; aRIR 1.78, 95%CI 1.20–2.62) when compared with the Australian population and within the cohort after adjusting for smoking. Laryngeal cancer was increased in Production workers (SIR 145, 95%CI 100–212).

**Conclusions:**

Increased rates of lung and laryngeal cancers were identified for male Production workers and lung cancer for Construction workers. These could be related to mine workplace exposures such as silica and diesel engine exhaust.

**Supplementary Information:**

The online version contains supplementary material available at 10.1007/s00420-025-02188-x.

## Objective

Coal mine workers are exposed to respirable coal mine dust (RCMD) and carcinogens, such as respirable crystalline silica (RCS), diesel engine emissions (DEE), and ultra-violet radiation from sunlight [1–4]. A recent meta-analysis identified 36 studies of cancer and mortality in male coal mine workers internationally [5]. There was a decreased risk of prostate cancer but no increased risk of lung cancer. However, many of the studies had short follow-up and were of cancer mortality rather than incidence. As many cancers are now treatable, mortality-only studies will likely underestimate the true cancer burden. None of the cohorts included individual smoking data or included female coal mine workers. The most recent follow-up period was to 2006.

This study aims to fill some of these knowledge gaps using mine worker data from Queensland, Australia. The coal mining industry in Queensland includes open-cut and underground mines handling mainly bituminous coal. Only 5 of the 106 mines had some semi anthracitic deposits and no lignite mines were included. In mid-2023, the industry employed 36,600 workers in open-cut or exploration sites and over 7,400 workers in underground mines [6].

A 1982 survey of Queensland coal miners identified increased risks of respiratory illness among current and retired coal mine workers [7]. Since this time, pre-employment medical assessments (including smoking status) have been mandated for all Queensland coal mine workers. After 1992, 5 yearly medical assessments were required and job titles were collected. Data from the assessments were submitted to, digitised and stored by the Queensland Government agency, Resources Safety and Health Queensland (RSHQ). More details about the cohort setup are available in a previous paper [8]. 

This paper examines differences in cancer incidence between workers allocated to different Work Categories based on their job titles and adjusted for smoking. Since detailed individual exposure data were unavailable, the Work Categories were used as proxies for variations in exposures across different areas of the mines.

## Methods

**Fig. 1:**
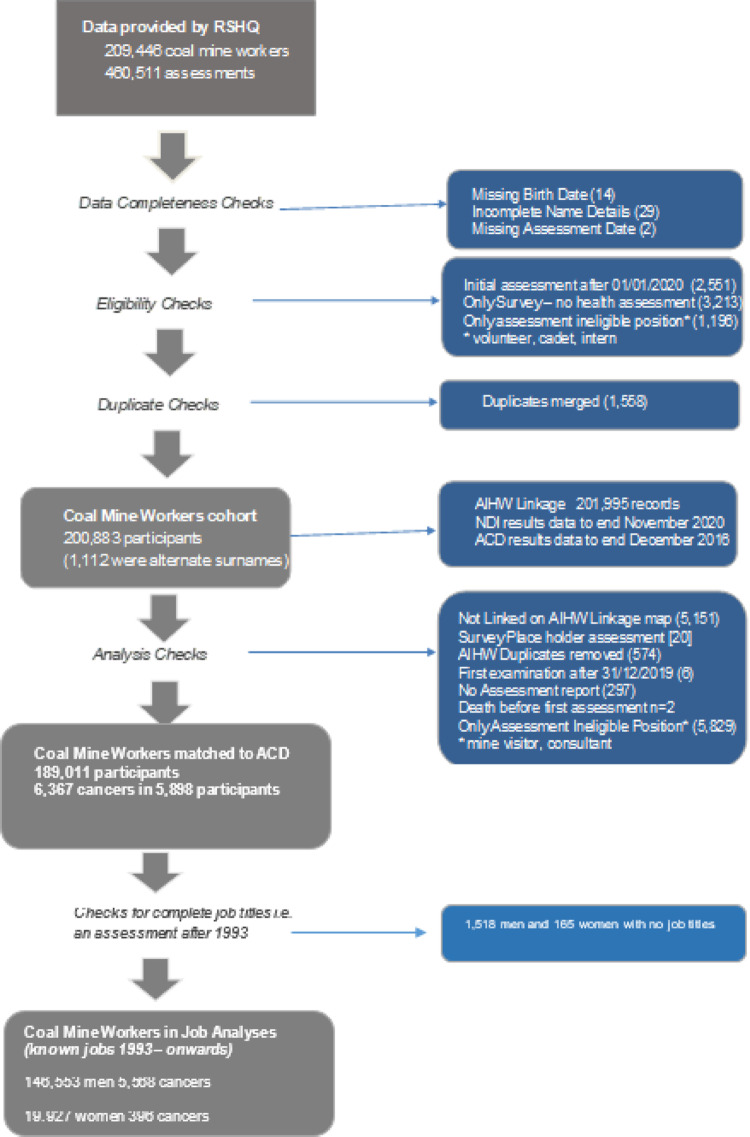
Cohort Structure showing numbers of coal mine workers and flow of data and exclusions (Individuals may have been excluded for more than one reason). Abbreviations: RSHQ = Resources Safety & Health Queensland, AIHW = Australian Institute of Health & Welfare, NDI = National Death Index, ACD = Australian Cancer Database.

This was a retrospective cohort study of cancer incidence of male and female workers who worked in the Queensland coal mines and had initial and/or 5-yearly assessments after 1992. At each medical assessment, identifying data were collected as well as smoking status, date of medical assessment, job title, mine name, and date of first assessment. These personal data were used to form the cohort (Fig. 1). No medical data were available from the assessments. While the RSHQ assessments began earlier than 1992, job title information was only collected from 1993 when 5-yearly assessments were mandated. The data presented in this paper, therefore, relate only to members of the cohort with a health assessment in or after 1993. More details of methods are presented in a previous paper relating to mortality by Work Category [9]. 

In order to assess cancer incidence by Work Category, the 49,490 unique free text job titles reported by the coal mine workers were categorized and coded into one of 42 Job Groups using documented inclusion and exclusion search terms by an experienced academic occupational hygienist (DG) and data manager (ADM). This included searching for misspellings, such as ACCOUNTANT or ACOUNTANT. Unusual job titles were checked using online sources, such as Peggy, SIS, MMU, and Moxy. The Job Groups were amalgamated to one of eight Work Categories: Production (including miners, operators), Maintenance (electricians, fitters, etc.), Administration, Unexposed Non-Office (e.g. control room, environmental services), Occasionally Exposed workers (e.g. engineers, surveyors), Exploration drillers, Construction or “Unclear” Jobs (Labourer, Cleaner, Supervisor, Truck Driver with no further information) (see Table 1). The job categorizations were checked with occupational hygienists employed by RSHQ and with worker and employer representatives on the Study Advisory Committee.

**Table 1:** Job groups in each work category

Work category	Job group
Unexposed Office	Administration
Unexposed Non-Office	Blue Collar (Not Exposed), Control Room, Environmental Services
Occasionally exposed	Engineer, Mine Services, Technical Services (Includes Geologist / Surveyor)
Maintenance- all	Maintenance – General Abrasive/Blast/Sand/Paint, Shutdown Maintenance, Belt Splicer, Boilermaker, Fitter, Diesel Fitter, Tyre Fitter, Electrician, Electrician (Auto), Industrial Cleaner
Production- all	Production – General, Blast Crew, Blast driller, CHPP Plant Operator, Laboratory, Driller (UG, Coal Seam), Explosion risk zone (ERZ) controller/ Deputy, Open cut Examiner Dragline, Secondary Support, Miner, Operator (Inc Mobile Operator, Production Operator, Production Truck Driver, Prestrip, Scraper, Production Support, Tunneller
Exploration	Driller (Exploration)
Construction	Civil Works, Construction
Work Category Unclear*	Labourer, Cleaner, Supervisor, Truck Driver with no further information

* Could not be classified into another Work Category, for example, Production or Maintenance

A small number of workers had changed jobs and therefore Work Category. They were included in each Work Category in the analyses. Workers were excluded from the Administration, Unexposed Non-Office and Occasionally exposed Work Categories if they had held jobs in other Work Categories (see Table 1).

Underground and open-cut workers were identified from several possible sources: the name of the mine or the type of mine where recorded (available for approximately 25% of workers). The job title (e.g., underground miner or open-cut examiner) and whether the employer had identified them as underground workers for the health assessment.

Some individuals were excluded: 300 workers were excluded because of missing personal information such as name or date of birth; 7025 individuals whose only job title was student, cadet, intern, or consultant were excluded as they were not likely to have been long-term coal mine workers; Visitors who held jobs such as arborist, film crew, weed control were also excluded. Additionally, workers were excluded from analyses if there was no job title recorded in any assessment (men *n* = 1518, women *n* = 165).

To identify incident cancers, the cohort was linked to the Australian Cancer Database (ACD) curated by the Australian Institute of Health and Welfare (AIHW) [10]. The ACD includes data about all new cancer cases diagnosed and/or treated in Australia and was complete from 01/01/1982 to 31/12/2016 at the time of the study. Linkage was conducted via the AIHW National Linkage Map (NLM), which only includes Australian residents registered in Medicare to receive Australian public health care. The small proportion of workers (3%) who could not be mapped to the NLM were also excluded from the analyses.

The cohort cancer incidence rates were compared to the national population rates to obtain cancer incidence ratios (SIR) for Work Categories and selected Job Groups (where there were sufficient numbers). The comparison data were obtained from the Australian Cancer Incidence and Mortality dataset [11]. The risks were calculated separately for men and women, and for specific types of cancers. Only primary cancers were included. The risk estimates were calculated using the Stata command *strate*.

A small proportion of workers (3%) could not be linked to the ACD through the National Linkage Map. Of these, 60% had their first assessment in or after 2010 so would have contributed relatively few person-years or cancer events.

The relative risks by Work Category, were calculated by comparing each Category to the rest of the cohort, providing relative cancer incidence ratios (RIRs). Smoking status (current, ex-smoker or never smoker) was used together with age and era (time period) in a Poisson regression model to adjust the calculated RIRs (reported as aRIR).

SIRs and aRIRs are reported together with their 95% confidence intervals (CI). Two-way interactions between age and smoking status were explored in the base Poisson regression model, and only statistically significant interactions (with p-value < 0.05) were included in the final models. Data were analyzed using Stata Statistical Package version 18 (StataCorp. 2023. Stata Statistical Software: Release 18. College Station, TX, StataCorp LLC).

The study was granted ethics approval including a waiver of individual consent by the Human Research Ethics Committees of Monash University (Study number 22729), the AIHW and state and territory Cancer Registries. Personal data are held by the Queensland Government and can be used for research and health surveillance purposes.

## Results

A total of 166,480 people (146,553 men and 19,927 women) were included in these analyses, including 5,103 men with 5,568, cancers and 373 women with 396 cancers (Table 2). The median age of workers at their first assessment varied across Work Categories (range 30–41 years among men and 30–36 years among women), and the average age at the end of follow-up was under 60. The majority of workers had worked either in the Maintenance or Production Work Categories.

**Table 2:** Cohort description, by work category

Work category	*N* (%) men or women	Number with cancers (%)	Median age at 1st assessment	% first assessment in or after 2010	Last Known Smoking status		
					Never smoked *n* (%)	Current smoker *n* (%)	Ex-smoker *n* (%)
ALL MEN *N* = 146,553	–	**5103 (3.5)**	**34.5**	**42.6**	**65,904 (45.3)**	**40,804 (28.0)**	**38,926 (26.7)**
ONLY Administration	11,339 (7.7)	409 (3.6)	41.1	56.0	6,393 (56.7)	1,953 (17.3)	2,923 (25.9)
ONLY Unexposed Non-Office	3,834 (2.6)	107 (2.8)	36.3	68.1	1,748 (46.0)	1,294 (34.0)	759 (20.0)
ONLY Occasionally exposed	9,522 (6.5)	195 (2.1)	33.1	55.9	6,413 (67.9)	1,478 (15.6)	1,561 (16.5)
EVER Maintenance- all	55,505 (37.9)	1,553 (2.8)	32.2	38.8	25,162 (45.6)	15,215 (27.6)	14,832 (26.9)
EVER Production	50,918 (34.7)	2,327 (4.6)	34.3	32.9	19,256 (38.0)	14,508 (28.7)	16,856 (33.3)
EVER Exploration driller	4,621 (3.2)	68 (1.5)	30.8	51.6	1,740 (37.9)	1,714 (37.3)	1,141 (24.8)
EVER Construction	8,435 (5.8)	217 (2.6)	34.7	51.9	3,290 (39.3)	3,204 (38.2)	1,886 (22.5)
*Unclear Work Category*							
EVER Labourer	5,445 (3.7)	104 (1.9)	30.3	38.6	1,925 (35.5)	2,213 (40.9)	1,280 (23.6)
EVER Cleaner	846 (0.6)	14 (1.7)	32.7	48.8	283 (33.8)	382 (45.6)	172 (20.6)
EVER Supervisor	5,477 (3.7)	265 (4.8)	36.9	25.5	2,001 (36.7)	1,378 (25.3)	2,076 (38.1)
EVER Truck Driver	7,209 (4.9)	364 (5.1)	40.2	35.8	2,584 (36.1)	2,259 (31.5)	2,319 (32.4)
**ALL WOMEN N = 19,927**	–	**373 (1.9)**	**32.8**	**53.3**	**10**,**465 (52.9)**	**5**,**251 (26.6)**	**4**,**054 (20.5)**
ONLY Administration	6,608 (33.2)	142 (2.2)	32.5	54.3	4,026 (61.4)	1,240 (18.9)	1,287 (19.6)
ONLY Unexposed Non-Office	1,721 (8.6)	28 (1.6)	35.2	68.6	822 (48.2)	582 (34.1)	302 (17.7)
ONLY Occasionally exposed	1,474 (7.5)	18 (1.2)	29.9	67.9	966 (66.2)	308 (21.1)	186 (12.7)
EVER Maintenance	1,503 (7.5)	20 (1.3)	31.5	55.9	683 (46.0)	472 (31.8)	331 (22.3)
EVER Production	5,362 (26.9)	85 (1.6)	32.2	48.1	2,450 (46.0)	1,477 (27.7)	1,402 (26.3)
EVER Exploration driller	34 (0.2)	< 6	30.9	50.0	17 (50.0)	10 (29.4)	7 (20.6)
EVER Construction	98 (0.5)	0	32.7	52.0	43 (43.9)	35 (35.7)	20 (20.4)
*Unclear Work Category*							
EVER Labourer	213 (1.1)	< 6	33.1	31.5	78 (37.1)	79 (37.6)	53 (25.2)
EVER Cleaner	2,529 (12.7)	60 (2.4)	36.0	46.7	957 (38.1)	1,066 (42.3)	488 (19.4)
EVER Supervisor	61 (0.3)	< 6	35.7	41.0	27 (44.3)	14 (23.0)	20 (32.8)
EVER Truck Driver	801 (4.0)	15 (1.9)	35.2	37.3	334 (42.0)	254 (31.9)	208 (26.1)

*Smoking status was not recorded for 1,076 workers (157 women and 919 men). Percentages were calculated for workers whose smoking status was known

Smoking status was available for more than 99% of the cohort. Approximately 45% of the cohort had never smoked, but the rate varied by Work Category. Never smokers comprised between 46% and 68% of male Administration, Unexposed non-office, Occasionally Exposed and Maintenance workers. In the other Work Categories, < 40% had never smoked. Women were more likely to be never-smokers than men (Table 2).

### Overall cancer incidence

**Fig. 2:**
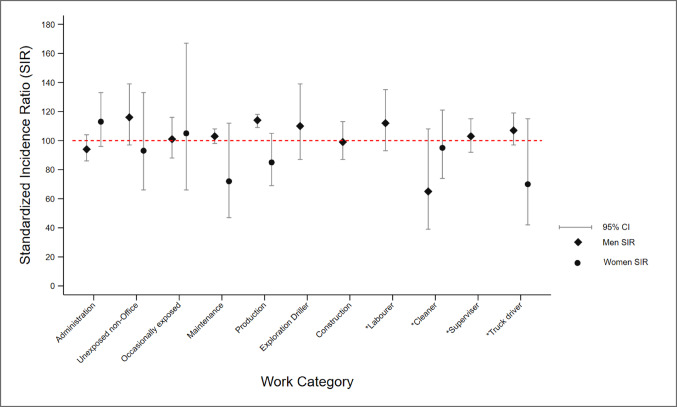
Summary of SIRs for all cancers combined, by sex and Work Category compared to Australian population, standardised for age and era. Asterisk (*) indicates Job Groups whose Work Categories were unclear

The overall cancer risk was increased for male Production workers, Unexposed non-office workers, Exploration drillers, and for Labourers and Truck drivers in the Unclear Work Category (Fig. 2) compared to the general population. For female coal mine workers, only Administration workers showed a possibly elevated risk of overall cancer when compared to the general population (Fig. 2).

When the risk of overall cancer in each Work Category for men was compared to the rest of the male cohort and adjusted for confounders, a significantly reduced risk of overall cancer was seen among those who had worked only in Administration (aRIR = 0.89, 95% CI 0.80–0.99) or ever worked in the Maintenance Work Category (aRIR = 0.92, 95% CI 0.87–0.98) or as Cleaners (aRIR = 0.56, 95% CI 0.33–0.95) (Table S3 shows adjusted and unadjusted risks). However, the risk of any cancer was 9% higher among those who had ever worked in the Production Work Category, compared with all the other workers (aRIR = 1.09, Table S3). All the other Work Categories had rates comparable to those of the rest of the male cohort.

Among female coal mine workers, risk of overall cancer was 30% higher among workers who were in the Only Administration Work Category compared with the rest of the female cohort (aRIR = 1.31, 95% CI 1.05–1.63), while cancer risk among the other Work Categories was comparable. (Table S6)

### Incidence of respiratory cancers

**Fig. 3:**
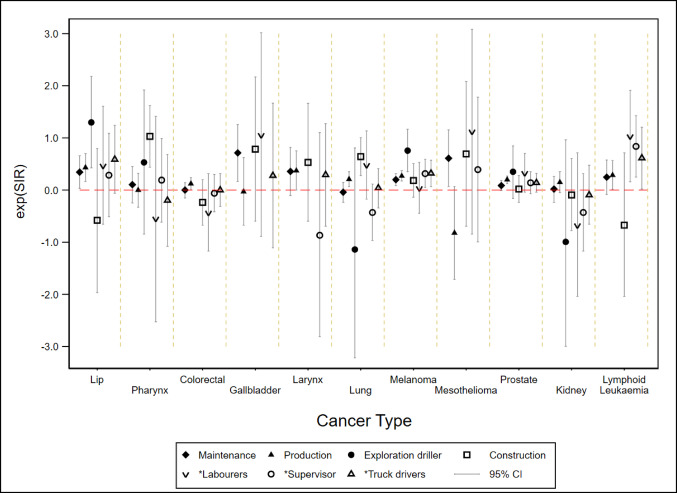
Comparison of male cohort cancer rates compared to Australian population rates, by work category, standardised for age and era. Asterisk (*) indicates Job Groups whose Work Categories were unclear

**Fig. 4:**
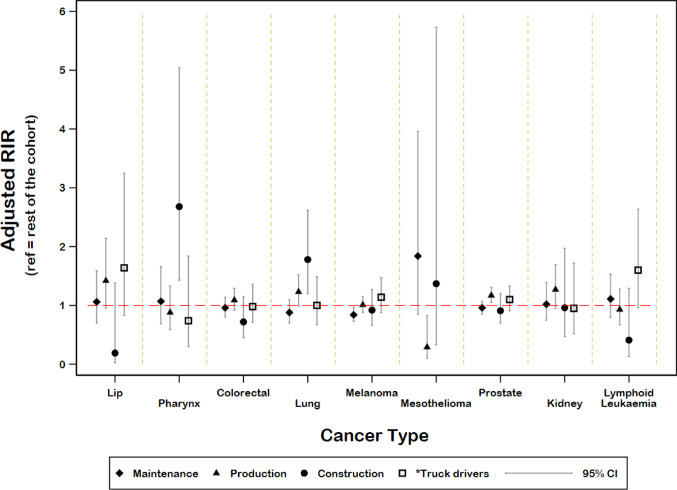
Risk of selected cancers for male Maintenance, Production, Construction Work Categories and Truck drivers compared with all other workers in the cohort not in that Work Category. Adjusted for age, era and smoking

Lung and Laryngeal cancers increased in some Work Categories. Compared to the general population, an increased risk of lung cancer incidence was seen in male Production workers and Construction workers, but not in other Work Categories (Fig. 3, Tables S1 and S2). An increased risk was also seen when these Work Categories were compared to the rest of the cohort after adjusting for confounders, notably smoking (Fig. 4 and Table S4 which present adjusted and unadjusted risks). Lung cancer incidence among men in the Maintenance Work Category was not elevated above the general population rates (Table S1).

Detailed Work Category comparisons for specific cancers among women were restricted by the small number of cancer cases among women in the cohort. For the two groups with a relatively large number of workers (Only Administration and Ever Production), the risk of lung cancer incidence was comparable to that of the general population, as well as to other female workers, adjusted and unadjusted risks are shown in Tables S5, S6 and S7.

Compared to the general population, laryngeal cancer incidence increased only for male Production workers (Fig. 3, Table S1) and not in any of the other Work Categories. Due to the small numbers of workers with laryngeal cancer, internal cohort comparisons were not performed.

### Incidence of other cancers

Increased incidence of cancer of the lip was seen for men who were Maintenance workers, Production workers and Exploration drillers compared to the general population and Truck drivers (Fig. 3, Table S1, and Table S2). In the smoking-adjusted analyses, the risk of lip cancer was increased for Production workers and Truck drivers when compared to the rest of the cohort (Fig. 4, Table S4).

The incidences of several other cancers were increased compared to the general Australian population, particularly for male workers ever in Production and Construction Work Categories (Fig. 3 and Table S1). Prostate cancer incidence was raised for male Maintenance and Production workers (Fig. 3) compared to the general population and workers who had ever worked in Production had higher prostate cancer rates than the rest of the cohort (Fig. 4).

There was an excess of lymphoid leukemia in men in the Production Work Category (Table S1), Labourers, Supervisors and Truck drivers (Table S2), and excess colorectal cancer incidence in men and women from the Production Work Category, compared to the general population (Fig. 3 and Table S1).

Melanoma was increased for men in several Work Categories (Fig. 3) and for women in Administration (SIR 1.76, 95%CI 1.10–2.81, *n* = 33) compared to the general Australian population. Gallbladder cancer was increased among male Maintenance workers (Table S1).

The incidence of mesothelioma was raised for men in Maintenance, Construction and Labourers compared to the general population and within the cohort. (Figures 2 and 3), respectively. There were no mesotheliomas among the female coal mine workers.

## Discussion

This study identified cancer risks separately for male and female coal mine workers and showed differences in cancer risk between male and female coal mine workers by job.

Compared to the general population, cancer risk varies across Work Categories and by sex. Male Production workers were more at risk of cancer than the general population and when compared to the rest of cohort after adjusting for smoking (Supplementary Table S8). Male Production workers showed an overall increased risk of cancer and specifically of lip, colorectal, larynx, lung, melanoma, prostate, urinary tract and lymphoid cancer. Male Maintenance workers have an increased risk of lip, gallbladder, melanoma, and mesothelioma. Administrative and the Occasionally exposed male workers had an increased risk of melanoma and mesothelioma. Male Exploration drillers had excess melanoma and lip cancers, Construction workers of pharyngeal and lung cancer Unexposed non-office workers of colorectal cancer, Truck drivers and Labourers of lymphoid leukemia. Melanoma risk was significantly increased in female Administration workers.

Lung cancer was higher among male Construction and Production workers compared to the rest of the cohort, after adjusting for era, age and smoking. Coal mine workers, particularly Production and Construction workers are exposed to respirable crystalline silica and diesel engine emissions, both of which are known carcinogens carcinogens [1, 2]. Underground coal miners cannot smoke at work so they may smoke less than the general population. A previous meta-analysis [5] did not show an increased risk of lung cancer in coal miners. However, lower smoking rates in coal mine workers may explain these findings, as case-control studies in which smoking was controlled showed higher risks for lung cancer than cohort studies with no smoking adjustment [5]. Investigation of the coal workers’ pneumoconiosis rates by Work Category could be informative.

The inclusion of many more recently exposed workers in the cohort may have diluted the observed risk because of the healthy worker effect. In other words, older workers may have been diagnosed with cancer and left the cohort before 1992. Alternatively, there may have been more dust exposure among Production workers in the earlier years [12]. 

Coal mine workers are exposed to coal mine dust, including RCS and DEE. However, exposure data were not available for specific mines nor for before 1992, so constructing an individual’s exposure history was not possible. Production workers are likely to be more exposed than Maintenance or Administration workers, and exposures are likely to have been higher, on average, for underground workers than for open-cut workers. Exposure data from the Queensland mines were reviewed by Cliff et al. [12]. The average exposure to respirable coal mine dust in the four Queensland Longwall mines in 1992–1994 varied between 1.3 and 3.5 mg/m^3^ [12]. Data from 11 Queensland underground mines from 1996 to 2001 identified mean 8 hour TWAs of between 1.59 and 3.24 mg/m^3^ (overall average 2.06 mg/m^3^). However, the data had a skewed distribution, with between 0 and 45% of the data from each mine exceeding the occupational exposure limit of 3 mg/m^3^ and reaching 15 mg/m^3^ [12]. Cliff pointed out that exposures were lower in New South Wales (NSW) coal mines during this period [12] and a cohort study of 23,630 male NSW coal miners found an all-cancer SIR of 82 (95%CI 73–92) [13], which was lower than that in this study. Between 2000 and 2017, the average yearly respirable coal mine dust (RCMD) exposure of Queensland underground production workers was between 0.75 and 2.75 mg/m^3^ [13]. It is likely that the average RCMD exposure was well below the occupational exposure limit of 3 mg/m^3^ for production workers who commenced work after 2000, but an increased incidence of lung cancer was still observed.

Between 2000 and 2017, the average yearly RCS exposure for Queensland underground production workers was usually between 0.01 and 0.06 mg/m^3^ [12]. Data from 11 of the 14 mines showed that annual exposures were usually below 0.025 mg/m^3^ between 2002 and 2017 [12]. Australian coal mines are thought to have thicker seams than many US mines, so the rock is less likely to be disturbed, leading to lower silica exposure [14]. Between 1999 and 2017, the average yearly RCS exposure for most Queensland open-cut production workers was somewhat lower than that for underground workers, between 0.01 and 0.015 mg/m^3^ [12]. Blast crew and dragline operators had an average exposure of approximately 0.02 mg/m^3^ RCS.

As the majority of the sites were open-cut, radon exposure is unlikely to be significant.

The risks of melanoma [3] and lip cancer [15] risks are likely related to sun exposure, particularly in Queensland. Other risk factors for lip cancer include age and male gender, poorer socio-economic circumstances, smoking and alcohol, sunlight exposure early in life and cumulatively, viral infections and immunosuppression [16]. In an earlier analysis [8] it was noted that the excess melanoma cases observed when comparing cohort rates to the Australian population were attenuated when the cohort rates were compared with the Queensland rates, that is, the elevated melanoma risks are related to the state of residence rather than occupation. Brown et al. found comparable risks of malignant melanoma between New South Wales (NSW) coal mine workers and the general population; however, for workers who started working in an open-cut mine, the risk was significantly increased. There was no increased risk of lip cancer in NSW coal miners [13]. 

The elevated prostate cancer incidence could be a result of diagnostic bias following increased screening in the working population, increased survival of patients with better general health, or earlier access to treatment. However, the within-cohort comparisons suggest that there is a real increase in the risk for Production workers.

Gallbladder cancer is a rare cancer that usually occurs in people over 80 years of age and is more common in women. There is an increase in gallbladder cancer among women and male maintenance workers. A recent systematic review identified possible associations of cholangiocarcinoma (the predominant type of gallbladder cancer) with the solvent 1,2-dichloropropane, asbestos, endocrine-disrupting compounds, and rotating shift work. [16} It is more likely that maintenance workers would be exposed to 1,2-dichloropropane and asbestos than would other coal mine workers. A meta analysis of 26 studies did not find a convincing association between gallbladder cancer and smoking or alcohol consumption [17]. 

Lymphoid leukaemia was increased for men in several Work Categories; Production, Truck drivers and Labourers. Chronic lymphocytic leukaemias, the largest group of lymphoid leukaemias, are now grouped with Non Hodgkin lymphoma (NHL) [18]. About 98% of the lymphoid leukaemias in this study were chronic lymphocytic leukaemias. The environmental causes of CLL are uncertain but may relate to living or working on a farm [19]. Large case control studies of NHL in Canada [20] and USA [21] did not identified an association with work as a miner. Brown et al. did not find an increased risk of lymphohaematopoietic cancers in NSW coal miners despite concern about an NHL cluster in a specific coal mine [13]. Gilman et al. [22] identified an increased odds ratio for leukaemia associated with more than 25 years working as an underground miner (as a proxy for higher electromagnetic field exposure). No other mine worker studies were identified that examined this outcome.

The study included multiple comparisons across eight job categories and several cancer types, for men and women (where possible) increasing the possibility of chance findings. In some cancer categories the numbers of cases were small. Repeating the findings in the future when more cases may have accumulated would be of interest. The results should be examined as a whole and considered in the context of findings from other cohorts [5]. 

The strength of this study is the complete enumeration of Queensland coal mine workers employed since 1993, including almost 20,000 women. Smoking data from at least one assessment were included in over 99% of the participants.

Cancer registration is mandatory in all Australian States and Territories, and registration, including for cancer, is virtually complete [23]. The cohort names were matched to those in the ACD, although the National Linkage Map is a probabilistic process. The availability of quality identifiers routinely collected over time by RSHQ records improves the probability of a correct match between cohort members and the ACD. Matching to the Australian national data was necessary because approximately 20% of the matches were identified in states other than Queensland. Coal mine workers may be Fly -in Fly-out from other states or retire to states other than Queensland, so matching only to the Queensland data would miss cancers.

The limitations of the study include the relative youth of the cohort and the short follow-up period, starting in or after 2003, for the majority of workers. The study was sufficiently powered to identify a significantly increased risk of overall cancer for female workers and major cancer categories for men in most Work Categories. There was a short period of follow-up for women, with small numbers of cancers reported, so caution should be used in interpreting these results. The latent period for cancers varies e.g. perhaps 10 years for leukaemia [24], around 10–15 years for many solid tumours [25] but can be 30–40 years for mesotheliomas [26]. The first assessment for half the cohort was in or after 2010 (Table 2), so it is probably too soon for many of the work-related cancers to manifest as the ACD data was only complete to the end of 2016.

The precise dates of employment as a coal mine worker were not known, only the dates of their first assessment and any subsequent assessments. Complete job histories were not accessible to the participants, the medical assessments did not include dates of change for jobs. Employment as a coal miner in other states or overseas has not been captured. Furthermore, it was not clear whether some individuals were underground workers, so there may be some misclassification of underground workers to open-cut. However, the classification as an underground worker is likely to be correct.

Additionally, the study could not consider individual genetic or lifestyle factors such as ethnicity, alcohol consumption, diet, non-occupational exposures, or occupational exposures experienced, for example, in previous jobs.

## Conclusions

Cancer rates were higher in this cohort of mainly bituminous coal miners than in the general population for many cancers, particularly among male Production workers. Lung cancer incidence rates were higher among Production and Construction workers compared to the rest of the cohort even after adjusting for smoking. Incidence of laryngeal cancer was also increased among Production workers. These higher risks could be related to workplace exposures such as coal mine dust, silica or DEE.

The incidence of lip cancer is higher in Production workers and Exploration drillers who work outside. Overall, the differences in cancer risks observed across Work Categories require further investigation to ensure workers in the jobs with higher risk are well protected. Additionally, smoking cessation, sun awareness programs, and cancer screening for melanoma and lung cancer, particularly for smokers in Production jobs, should be considered for workers in this industry.

## Supplementary Information

Below is the link to the electronic supplementary material.

Supplementary Material 1

## Data Availability

Health data have been obtained from an Australian national repository and are not available to other researchers. Coal mine worker identifiers were obtained from Queensland government Department RSHQ. Original data are not available as a result of Australian privacy legislation and terms of our Ethics approvals.
