# Supplementary material for: Does your job really matter? Job-specific cancer incidence among a cohort coal mine workers in Queensland, Australia
Source: Int Arch Occup Environ Health. 2026 Jan 7;99(1):7. doi: 10.1007/s00420-025-02188-x (PMC12779655; doi:10.1007/s00420-025-02188-x)
Supplement: Supplementary file 1 — Supplementary Material 1 [file 420_2025_2188_MOESM1_ESM.docx]

# Does your job really matter? Job-specific cancer incidence among a cohort coal mine workers in Queensland, Australia

# Supplementary Tables

**Table S1: Standardized Incidence Ratios for male coal mine workers by Work Category, compared to the Australian population standardised for age and era**

| **Cancer Categories** | ICD-10 codes | **Maintenance**  **N=55,505; PY=532,772** | | | **Production**  ***N=50,918; PY=550,726*** | | | **Ever Exploration driller**  **N=4,621; PY=36,068** | | | **Ever Construction**  **N=8,435; PY=67,179** | | |
| --- | --- | --- | --- | --- | --- | --- | --- | --- | --- | --- | --- | --- | --- |
|  |  | **O** | **E** | **SIR (95% CI)** | **O** | **E** | **SIR (95% CI)** | **O** | **E** | **SIR (95% CI)** | **O** | **E** | **SIR (95% CI)** |
| **All cancers** | C00-C97,  D45-D46, D47.1, D47.3-D47.5 | 1680 | 1633 | 103 (98 - 108) | 2522 | 2220 | 114 (109 - 118) | 71 | 65 | 110 (87 - 139) | 227 | 229 | 99 (87 - 113) |
| **Lip, Oral Cavity and Pharynx** | C00-C14 | 100 | 90 | 112 (92 - 136) | 130 | 117 | 111 (93 - 132) | 9 | 4 | 228 (119 - 439) | 16 | 12 | 130 (80 - 213) |
| Lip | C00 | 39 | 28 | 141 (103 - 193) | 54 | 35 | 154 (118 - 201) | <6 |  | 366 (153 - 884) | <6 |  | 56 (14 - 222) |
| Pharynx | C09-C14 | 31 | 28 | 111 (78 - 157) | 37 | 37 | 100 (72 - 138) | <6 |  | 170 (43 - 681) | 11 | 4 | 280 (155 - 505) |
| **Digestive Organs** | C15-C25 | 299 | 310 | 96 (86 - 108) | 434 | 432 | 100 (91 – 110) | <6 |  | 17 (4 - 69) | 37 | 44 | 84 (61 - 116) |
| Oesophagus | C15 | 18 | 21 | 86 (54 - 136) | 35 | 30 | 116 (84 - 162) | 0 |  |  | <6 |  | 100 (32 - 311) |
| Stomach | C16 | 27 | 30 | 91 (63 - 133) | 30 | 42 | 72 (51 - 103) | <6 |  | 92 (13 - 651) | <6 |  | 72 (23 - 224) |
| Colorectal | C18-C21 | 181 | 181 | 100 (86 - 115) | 285 | 253 | 113 (100 - 127) | 0 |  |  | 20 | 25 | 78 (51 - 122) |
| Colon | C18 | 98 | 100 | 98 (81 - 120) | 159 | 139 | 114 (98 - 133) | 0 |  |  | 12 | 14 | 86 (49 - 151) |
| Rectum | C19 -C20 | 81 | 77 | 106 (85 - 131) | 122 | 107 | 114 (96 - 137) | 0 |  |  | 8 | 11 | 74 (37 - 149) |
| Liver | C22 | 24 | 34 | 71 (48 - 106) | 24 | 45 | 53 (35 - 79) | 0 |  |  | 6 | 5 | 123 (55 - 273) |
| Gallbladder | C23-C24 | 13 | 6 | 204 (118 - 351) | 9 | 9 | 97 (51 - 187) | 0 |  |  | <6 |  | 219 (55 - 875) |
| Pancreas | C25 | 26 | 30 | 88 (60 - 129) | 34 | 42 | 82 (58 - 114) | <6 |  | 96 (14 - 681) | <6 |  | 47 (12 - 188) |
| **Respiratory & Intrathoracic Organs** | C30-C38 | 130 | 126 | 103 (87 - 122) | 229 | 186 | 123 (108 - 140) | <6 |  | 52 (13 - 206) | 32 | 18 | 178 (126 - 252) |
| Larynx | C32 | 18 | 13 | 143 (90 - 227) | 27 | 19 | 145 (100 – 212) | 0 |  |  | <6 |  | 170 (55 - 528) |
| Lung | C33-C34 | 103 | 107 | 96 (79 - 116) | 197 | 160 | 123 (107 - 142) | <6 |  | 32 (4 - 224) | 29 | 15 | 189 (131 - 272) |
| **Melanoma** | C43 | 281 | 230 | 122 (109 - 137) | 387 | 295 | 131 (119 - 145) | 23 | 11 | 213 (142 - 321) | 37 | 31 | 119 (86 - 165) |
| **Mesothelioma** | C45 | 13 | 7 | 184 (107 - 317) | <6 |  | 44 (18 - 107) | 0 |  |  | <6 |  | 200 (50 - 800) |
| **Breast** | C50 | <6 |  | 141 (53 - 376) | <6 |  | 50 (13 - 200) | 0 |  |  | <6 |  | 250 (35 - 1771) |
| **Male Reproductive Organs** | C60-C63 | 473 | 460 | 103 (94 - 113) | 770 | 646 | 119 (111 - 128) | 19 | 15 | 123 (78 - 192) | 67 | 66 | 101 (79 - 128) |
| Prostate | C61 | 430 | 395 | 109 (99 - 120) | 712 | 584 | 122 (113 - 131) | 15 | 11 | 142 (85 - 233) | 59 | 58 | 101 (79 - 131) |
| Testis | C62 | 42 | 61 | 69 (51 - 93) | 54 | 58 | 94 (72 - 123) | <6 |  | 85 (32 - 225) | 8 | 8 | 104 (52 - 209) |
| **Urinary Tract** | C64-C68 | 94 | 91 | 103 (84 - 126) | 148 | 125 | 118 (101 - 139) | <6 |  | 84 (27 - 261) | 12 | 13 | 93 (53 - 164) |
| Kidney | C64 | 63 | 62 | 102 (79 - 130) | 95 | 82 | 116 (95 - 142) | <6 |  | 37 (5 - 262) | 8 | 9 | 91 (46 - 183) |
| Bladder | C67 | 25 | 25 | 99 (67 - 146) | 46 | 38 | 121 (91 - 162) | <6 |  | 271 (68 - 1082) | <6 |  | 83 (27 - 258) |
| **Brain and Other CNS** | C70-C72 | 34 | 37 | 93 (67 - 130) | 52 | 45 | 117 (89 - 153) | <6 |  | 105 (26 - 418) | <6 |  | 41 (10 - 164) |
| Brain | C71 | 34 | 35 | 97 (70 - 136) | 51 | 43 | 119 (91 - 157) | <6 |  | 110 (27 - 438) | <6 |  | 43 (11 - 171) |
| **Thyroid & Other Endocrine Glands** | C73-C75 | 28 | 33 | 85 (59 - 124) | 41 | 38 | 109 (80 - 148) | <6 |  | 152 (49 - 471) | <6 |  | 22 (3 - 159) |
| Thyroid | C73 | 27 | 30 | 89 (61 - 129) | 39 | 35 | 112 (82 - 153) | <6 |  | 109 (27 - 436) | <6 |  | 24 (3 - 170) |
| **Unknown Site** | C77-C80, C97 | 21 | 22 | 95 (62 - 146) | 36 | 32 | 111 (80 - 154) | <6 |  | 139 (20 - 987) | <6 |  | 66 (17 - 264) |
| **Lymphoid, Haematopoietic + Related Tissue** | C81-C96, D46 | 153 | 167 | 92 (78 - 107) | 194 | 214 | 91 (79 - 105) | <6 |  | 38 (12 - 119) | 10 | 23 | 44 (24 - 82) |
| Hodgkin | C81 | 8 | 17 | 46 (23 - 92) | 13 | 17 | 75 (44 - 130) | 0 |  |  | <6 |  | 91 (23 - 362) |
| Non-Hodgkin Lymphoma | C82- C86 | 72 | 72 | 100 (79 - 126) | 80 | 94 | 85 (68 - 106) | <6 |  | 62 (16 - 248) | <6 |  | 30 (10 - 94) |
| Diffuse Non-Hodgkin Lymphoma | C83 | 32 | 35 | 90 (64 - 128) | 34 | 46 | 73 (52 - 102) | 0 |  |  | <6 |  | 41 (10 - 165) |
| Multiple Myeloma | C90 | 14 | 20 | 70 (41 - 118) | 28 | 28 | 99 (68 - 143) | <6 |  | 142 (20 - 1009) | <6 |  | 69 (17 - 277) |
| Leukaemia | C91-C95 | 58 | 52 | 112 (86 - 145) | 70 | 67 | 104 (82 - 132) | 0 |  |  | <6 |  | 42 (14 - 131) |
| Lymphoid Leukaemia | C91 | 36 | 28 | 128 (92 - 178) | 50 | 38 | 133 (101 - 176) | 0 |  |  | <6 |  | 51 (13 - 204) |
| Myeloid Leukaemia | C92 | 16 | 20 | 81 (49 - 132) | 14 | 24 | 58 (34 - 98) | 0 |  |  | 0 |  |  |
| **Other Cancers (**C26, C39, C40-41, C44, C46, C47-C49, C69, C76, D45- D47) |  | 50 | 61 | 82 (62 - 109) | 94 | 79 | 119 (97 - 146) | <6 |  | 145 (54 - 387) | 8 | 8 | 96 (48 - 192) |
| Myelodysplastic Syndrome (MDS) | D46 | <6 |  | 55 (21 - 148) | 13 | 11 | 120 (70 - 207) | <6 |  | 939 (235 - 3754) | 0 |  |  |
| Connective tissue | D47-D49 | 11 | 14 | 76 (42 - 138) | 17 | 17 | 98 (61 - 158) | 0 |  |  | <6 |  | 104 (26 - 415) |

**Table S2 Standardized Incidence Ratios for male coal mine workers by Job Groups in the Unclear Work Categories, compared to the Australian population standardised for age and era**

| **Cancer Categories** | **Labourer**  **N=5,445; PY=47,754** | | | **Supervisor**  **N=5,447; PY=64,670** | | | **Truck Driver**  **N=7,209; PY=67,813** | | |
| --- | --- | --- | --- | --- | --- | --- | --- | --- | --- |
|  | **O** | E | SIR (95% CI) | O | E | SIR (95% CI) | O | E | SIR (95% CI) |
| **All cancers** | 112 | 100 | 112 (93 - 135) | 291 | 283 | 103 (92 - 115) | 380 | 354 | 107 (97 - 119) |
| **Lip, Oral Cavity and Pharynx** | 8 | 6 | 140 (70 - 280) | 18 | 15 | 117 (74 - 186) | 24 | 18 | 131 (88 - 195) |
| Lip | <6 |  | 160 (52 - 497) | 6 | 4 | 133 (60 - 297) | 9 | 5 | 180 (94 - 347) |
| Pharynx | <6 |  | 58 (8 - 410) | 6 | 5 | 121 (54 - 269) | <6 |  | 82 (34 - 198) |
| **Digestive Organs** | 23 | 18 | 127 (84 - 191) | 45 | 55 | 82 (61 - 109) | 65 | 70 | 93 (73 - 119) |
| Oesophagus | <6 |  | 87 (12 - 619) | <6 |  | 103 (39 - 273) | 7 | 5 | 141 (67 - 295) |
| Stomach | <6 |  | 59 (8 - 417) | <6 |  | 57 (18 - 176) | 8 | 7 | 121 (60 - 242) |
| Colorectal | 7 | 11 | 65 (31 - 137) | 30 | 32 | 94 (66 - 135) | 40 | 40 | 100 (73 – 137) |
| Colon | <6 | <6 | 85 (35 - 204) | 17 | 17 | 98 (61 - 158) | 20 | 22 | 91 (59 - 142) |
| Rectum | <6 | <6 | 44 (11 - 177) | 12 | 14 | 89 (50 - 156) | 20 | 17 | 118 (76 - 183) |
| Liver | **9** | 2 | 449 (234 - 864) | <6 |  | 82 (34 - 196) | <6 |  | 25 (6 - 102) |
| Gallbladder | <6 |  | 288 (41 - 2042) | 0 |  |  | <6 |  | 132 (33 - 529) |
| Pancreas | <6 |  | 60 (8 - 424) | <6 |  | 37 (9 - 149) | <6 |  | 73 (30 - 175) |
| **Respiratory and Intrathoracic Organs** | 10 | 7 | 150 (81 - 278) | 15 | 23 | 64 (39 - 107) | 31 | 30 | 103 (72 - 146) |
| Larynx | 0 |  |  | <6 |  | 42 (6 - 301) | <6 |  | 134 (50 - 357) |
| Lung | 9 | 6 | 162 (84 - 311) | 13 | 20 | 65 (38 - 112) | 27 | 26 | 104 (71 - 151) |
| **Melanoma** | 16 | 15 | 104 (64 - 170) | 52 | 38 | 137 (105 - 180) | 61 | 44 | 138 (107 - 177) |
| **Mesothelioma** | <6 |  | 308 (43 - 2184) | <6 |  | 148 (37 - 593) | 0 |  |  |
| **Breast** | 0 |  |  | 0 |  |  | 0 |  |  |
| **Male Reproductive Organs** | 32 | 26 | 122 (86 - 172) | 98 | 82 | 119 (98 - 145) | 122 | 108 | 113 (95 - 135) |
| Prostate | 28 | 20 | 139 (96 - 202) | 87 | 76 | 115 (93 - 142) | 116 | 101 | 115 (96 - 138) |
| Testis | <6 |  | 67 (25 - 178) | 11 | 6 | 172 (95 - 311) | <6 |  | 64 (24 - 171) |
| **Urinary Tract** | <6 |  | 37 (9 - 147) | 14 | 16 | 87 (52 - 146) | 13 | 20 | 64 (37 - 111) |
| Kidney | <6 |  | 51 (13 – 204) | 7 | 11 | 65 (31 - 137) | 12 | 13 | 91 (52 - 161) |
| Bladder | 0 |  |  | 7 | 5 | 148 (71 - 310) | 0 |  |  |
| **Brain and Other CNS** | <6 |  | 76 (19 - 303) | <6 |  | 36 (9 - 143) | 8 | 6 | 124 (62 - 248) |
| Brain | <6 |  | 79 (20 - 317) | <6 |  | 37 (9 - 149) | 8 | 6 | 129 (65 - 258) |
| **Thyroid and Other Endocrine Glands** | <6 |  | 39 (5 - 276) | <6 |  | 84 (32 - 224) | <6 |  | 55 (18 - 170) |
| Thyroid | <6 |  | 42 (6 - 298) | <6 |  | 90 (34 - 240) | <6 |  | 59 (19 - 182) |
| **Unknown Site** | <6 |  | 169 (42 - 677) | <6 |  | 99 (37 - 263) | <6 |  | 60 (19 - 186) |
| **Lymphoid, Haematopoietic + Related Tissue** | 12 | 11 | 106 (60 - 187) | 29 | 27 | 108 (75 - 155) | 35 | 33 | 108 (77 - 150) |
| Hodgkin | <6 |  | 121 (30 - 485) | <6 |  | 51 (7 - 363) | <6 |  | 47 (7 - 337) |
| Non-Hodgkin Lymphoma | <6 |  | 64 (21 - 198) | 9 | 12 | 75 (39 - 144) | 11 | 14 | 76 (42 - 137) |
| Diffuse Non-Hodgkin Lymphoma | 0 |  |  | <6 |  | 17 (2 - 120) | 7 | 7 | 97 (46 - 204) |
| Multiple Myeloma | <6 |  | 176 (44 - 705) | <6 |  | 136 (57 - 327) | <6 |  | 106 (44 - 256) |
| Leukaemia | <6 |  | 144 (60 - 345) | 13 | 8 | 154 (90 - 265) | 18 | 10 | 175 (110 - 278) |
| Lymphoid Leukaemia | <6 |  | 281 (117 - 676) | 11 | 5 | 231 (128 - 416) | 11 | 6 | 185 (102 - 334) |
| Myeloid Leukaemia | 0 |  |  | <6 |  | 33 (5 - 236) | 6 | 3 | 173 (78 - 385) |
| **Other Cancers** | <6 |  | 74 (24 - 231) | 8 | 10 | 80 (40 - 161) | 15 | 12 | 125 (75 - 207) |
| Myelodysplastic Syndrome (MDS) | <6 |  | 260 (37 - 1843) | <6 |  | 75 (11 - 535) | <6 |  | 114 (28 - 455) |
| Connective tissue | <6 |  | 94 (13 - 670) | <6 |  | 92 (23 - 368) | <6 |  | 79 (20 - 317) |

Table S3: Relative Incidence Ratios (RIRs) for overall cancers among men, for each Work Categories compared with the rest of the male cohort not in that Work Category

| Category | Number with at least one cancer (%) | Unadjusted  RIR (95% CI) | Adjusted*  RIR (95% CI) |
| --- | --- | --- | --- |
| ONLY Administration | 409 (3.6) | 1.29 (1.17 - 1.42) | 0.89 (0.80 - 0.99) |
| ONLY Unexposed Non-Office | 106 (2.8) | 1.14 (0.94 - 1.38) | 1.03 (0.84 - 1.26) |
| ONLY Occasionally exposed | 195 (2.0) | 0.70 (0.61 - 0.81) | 0.98 (0.85 - 1.14) |
| EVER Maintenance - all | 1,553 (2.8) | 0.67 (0.64 - 0.72) | 0.92 (0.87 - 0.98) |
| EVER Production - all | 2,327 (4.6) | 1.23 (1.17 - 1.30) | 1.09 (1.03 - 1.15) |
| EVER Exploration Driller | 68 (1.5) | 0.49 (0.39 - 0.62) | 1.02 (0.80 - 1.30) |
| EVER Construction - all | 217 (2.6) | 0.85 (0.74 - 0.97) | 0.93 (0.81 - 1.07) |
| Ever Labourer ** | 104 (1.9) | 0.57 (0.47 - 0.69) | 1.04 (0.85 - 1.26) |
| Ever Cleaner ** | 14 (1.7) | 0.55 (0.33 - 0.93) | 0.56 (0.33 - 0.95) |
| Ever Supervisor ** | 265 (4.8) | 1.10 (0.97 - 1.24) | 0.94 (0.83 - 1.06) |
| Ever Truck driver ** | 364 (5.0) | 1.47 (1.32 - 1.63) | 1.06 (0.95 - 1.18) |

* Adjusted for era of first examination, age, smoking status, age * era interaction & age * smoking status interaction

****** Unknown Work Category.

Table S4: Relative Incidence Ratios for male coal mine workers by Work Category compared with the rest of the male cohort not in that Work Category (adjusted for age and smoking)

| **Cancer Categories** | **Ever Maintenance** | | **Ever Production** | | **Ever Construction** | | **Ever Truck driver*** | |
| --- | --- | --- | --- | --- | --- | --- | --- | --- |
|  | **Unadjusted** | **Adjusted *** | **Unadjusted** | **Adjusted *** | **Unadjusted** | **Adjusted *** | **Unadjusted** | **Adjusted *** |
| **Lip, Oral Cavity and Pharynx** | 0.83 (0.65 - 1.06) | 1.04 (0.81 - 1.34) | 1.21 (0.96 - 1.53) | 1.07 (0.85 - 1.36) | 1.16 (0.70 - 1.92) | 1.09 (0.65 - 1.85) | 1.78 (1.17 - 2.70) | 1.30 (0.85 - 1.98) |
| Lip | 0.96 (0.65 - 1.43) | 1.06 (0.70 - 1.59) | 1.65 (1.12 - 2.43) | 1.42 (0.95 - 2.14) | 0.39 (0.10 - 1.56) | 0.19 (0.03 - 1.38) | 1.85 (0.93 - 3.66) | 1.64 (0.83 - 3.25) |
| Pharynx | 0.79 (0.51 - 1.22) | 1.07 (0.69 - 1.66) | 0.99 (0.65 - 1.50) | 0.88 (0.59 - 1.33) | 2.61 (1.39 - 4.90) | 2.68 (1.43 - 5.04) | 1.09 (0.44 - 2.69) | 0.74 (0.30 - 1.84) |
| **Digestive Organs** | 0.70 (0.61 - 0.80) | 1.02 (0.88 - 1.17) | 1.17 (1.03 - 1.32) | 1.01 (0.88 - 1.15) | 0.78 (0.56 - 1.08) | 0.82 (0.58 - 1.16) | 1.35 (1.04 - 1.74) | 0.97 (0.75 - 1.25) |
| Oesophagus | 0.58 (0.34 - 1.00) | 0.85 (0.49 - 1.48) | 1.64 (1.01 - 2.64) | 1.41 (0.84 - 2.34) | 0.91 (0.29 - 2.90) | 0.67 (0.16 - 2.72) | 2.25 (1.03 - 4.92) | 1.64 (0.75 - 3.61) |
| Stomach | 0.89 (0.55 - 1.42) | 1.35 (0.83 - 2.18) | 1.00 (0.63 - 1.58) | 0.83 (0.49 - 1.40) | 0.81 (0.26 - 2.57) | 0.85 (0.27 - 2.71) | 2.30 (1.11 - 4.79) | 1.52 (0.72 - 3.21) |
| Colorectal | 0.67 (0.56 - 0.79) | 0.96 (0.80 - 1.14) | 1.28 (1.09 - 1.50) | 1.09 (0.92 - 1.29) | 0.66 (0.42 - 1.03) | 0.72 (0.45 - 1.15) | 1.32 (0.95 - 1.82) | 0.98 (0.71 - 1.36) |
| Liver | 0.69 (0.43 - 1.11) | 1.06 (0.65 - 1.71) | 0.65 (0.40 - 1.05) | 0.69 (0.43 - 1.13) | 1.60 (0.70 - 3.68) | 1.44 (0.62 - 3.30) | 0.50 (0.12 - 2.04) | 0.30 (0.07 - 1.22) |
| Pancreas | 0.71 (0.45 - 1.12) | 1.08 (0.67 - 1.73) | 1.02 (0.66 - 1.57) | 0.85 (0.55 - 1.31) | 0.47 (0.12 - 1.93) | 0.52 (0.12 - 2.21) | 1.22 (0.49 - 3.01) | 0.85 (0.34 - 2.11) |
| **Respiratory & Intrathoracic Organs** | 0.62 (0.51 - 0.76) | 0.94 (0.77 - 1.16) | 1.50 (1.25 - 1.81) | 1.19 (0.97 - 1.45) | 1.48 (1.03 - 2.12) | 1.60 (1.11 - 2.31) | 1.42 (0.98 - 2.04) | 0.96 (0.67 - 1.39) |
| Lung | 0.57 (0.46 - 0.72) | 0.88 (0.70 - 1.10) | 1.58 (1.30 - 1.93) | 1.23 (0.99 - 1.52) | 1.59 (1.09 - 2.33) | 1.78 (1.20 - 2.62) | 1.46 (0.99 - 2.16) | 1.00 (0.67 - 1.49) |
| **Melanoma** | 0.70 (0.61 - 0.81) | 0.84 (0.73 - 0.97) | 1.10 (0.97 - 1.26) | 1.01 (0.88 - 1.15) | 0.82 (0.59 - 1.14) | 0.92 (0.66 - 1.27) | 1.39 (1.07 - 1.80) | 1.14 (0.88 - 1.47) |
| **Mesothelioma** | 1.14 (0.56 - 2.33) | 1.84 (0.85 - 3.96) | 0.29 (0.11 - 0.75) | 0.29 (0.10 - 0.83) | 1.34 (0.32 - 5.62) | 1.37 (0.33 - 5.73) | --- | --- |
| **Male Reproductive Organs** | 0.63 (0.57 - 0.71) | 0.93 (0.84 - 1.04) | 1.32 (1.19 - 1.45) | 1.16 (1.05 - 1.28) | 0.81 (0.64 - 1.04) | 0.94 (0.73 - 1.21) | 1.55 (1.29 - 1.86) | 1.08 (0.89 - 1.30) |
| Prostate | 0.63 (0.56 - 0.70) | 0.96 (0.85 - 1.07) | 1.34 (1.21 - 1.48) | 1.17 (1.05 - 1.31) | 0.78 (0.60 - 1.01) | 0.91 (0.70 - 1.20) | 1.61 (1.33 - 1.94) | 1.10 (0.91 - 1.33) |
| Testis | 0.75 (0.52 - 1.09) | 0.70 (0.48 - 1.01) | 1.06 (0.75 - 1.51) | 1.15 (0.81 - 1.62) | 1.28 (0.62 - 2.61) | 1.20 (0.59 - 2.45) | 0.61 (0.23 - 1.65) | 0.66 (0.24 - 1.80) |
| **Urinary Tract** | 0.71 (0.56 - 0.91) | 0.98 (0.77 - 1.26) | 1.41 (1.13 - 1.77) | 1.23 (0.97 - 1.55) | 0.80 (0.45 - 1.43) | 0.91 (0.51 - 1.62) | 0.86 (0.50 - 1.50) | 0.61 (0.35 - 1.08) |
| Kidney | 0.77 (0.57 -1.03) | 1.02 (0.75 - 1.39) | 1.42 (1.07 - 1.88) | 1.27 (0.95 - 1.69) | 0.84 (0.41 - 1.71) | 0.96 (0.47 - 1.97) | 1.28 (0.71 - 2.29) | 0.95 (0.52 - 1.72) |
| Bladder | 0.56 (0.35 - 0.88) | 0.81 (0.51 - 1.28) | 1.38 (0.92 - 2.05) | 1.15 (0.74 - 1.79) | 0.63 (0.20 - 1.98) | 0.70 (0.22 - 2.21) |  |  |
| **Brain and Other CNS** | 0.65 (0.43 - 0.96) | 0.76 (0.51 - 1.14) | 1.20 (0.83 - 1.72) | 1.13 (0.76 - 1.69) | 0.34 (0.08 - 1.37) | 0.36 (0.09 - 1.46) | 1.41 (0.69 - 2.90) | 1.21 (0.58 - 2.51) |
| Brain | 0.66 (0.44 - 0.99) | 0.79 (0.52 - 1.18) | 1.19 (0.82 - 1.72) | 1.14 (0.76 - 1.70) | 0.34 (0.09 - 1.39) | 0.37 (0.09 - 1.49) | 1.44 (0.70 - 2.95) | 1.22 (0.59 - 2.54) |
| **Thyroid and Other Endocrine Glands** | 0.69 (0.44 - 1.08) | 0.80 (0.51 - 1.24) | 1.20 (0.80 - 1.81) | 1.14 (0.75 - 1.73) | 0.21 (0.03 - 1.53) | 0.23 (0.03 - 1.66) | 0.65 (0.21 - 2.05) | 0.56 (0.18 - 1.78) |
| Thyroid | 0.71 (0.45 - 1.12) | 0.82 (0.52 - 1.29) | 1.22 (0.80 - 1.85) | 1.18 (0.76 - 1.81) | 0.23 (0.03 - 1.62) | 0.24 (0.03 - 1.73) | 0.69 (0.22 - 2.18) | 0.58 (0.18 - 1.85) |
| **Unknown Site** | 0.58 (0.35 - 0.96) | 0.89 (0.54 - 1.47) | 1.28 (0.82 – 2.00) | 0.95 (0.60 - 1.50) | 0.51 (0.13 - 2.08) | 0.61 (0.14 - 2.58) | 0.77 (0.24 - 2.44) | 0.57 (0.18 - 1.82) |
| **Lymphoid, Haematopoietic + Related Tissue** | 0.79 (0.65 - 0.96) | 1.05 (0.86 - 1.28) | 1.06 (0.88 - 1.27) | 0.94 (0.77 - 1.14) | 0.44 (0.24 - 0.82) | 0.49 (0.26 - 0.92) | 1.57 (1.11 - 2.22) | 1.16 (0.81 - 1.66) |
| Hodgkin | 0.66 (0.29 - 1.52) | 0.70 (0.30 - 1.64) | 1.39 (0.65 - 2.95) | 1.49 (0.71 - 3.12) | 1.56 (0.37 - 6.57) | 1.37 (0.33 - 5.68) | 0.74 (0.10 - 5.46) | 0.71 (0.10 - 5.15) |
| Non-Hodgkin Lymphoma | 0.96 (0.71 - 1.28) | 1.22 (0.91 - 1.65) | 1.05 (0.79 - 1.41) | 0.89 (0.66 - 1.20) | 0.31 (0.10 - 0.97) | 0.37 (0.12 - 1.14) | 1.18 (0.64 - 2.16) | 0.97 (0.52 - 1.80) |
| Leukaemia | 0.80 (0.58 - 1.11) | 1.11 (0.80 - 1.53) | 1.06 (0.78 - 1.44) | 0.93 (0.67 - 1.28) | 0.35 (0.11 - 1.10) | 0.41 (0.13 - 1.29) | 2.3 (1.41 - 3.75) | 1.60 (0.96 - 2.64) |
| **Other Cancers** | 0.60 (0.43 - 0.83) | 0.80 (0.57 - 1.11) | 1.56 (1.17 - 2.09) | 1.37 (1.02 - 1.85) | 0.89 (0.44 - 1.82) | 1.01 (0.50 - 2.05) | 1.73 (1.02 - 2.93) | 1.34 (0.79 - 2.27) |

* Could not be classified to another Work Category e.g. Production or Maintenance

**Table S5: Standardised Incidence Ratios for female coal mine workers who by Work Category, compared to the Australian population standardised for age and era**

| **Cancer Categories** | **Only Administration** | | | **Ever Production**  **N= 5,361; PY= 41,637** | | |
| --- | --- | --- | --- | --- | --- | --- |
|  | **O** | **E** | **SIR (95% CI)** | **O** | **E** | **SIR (95% CI)** |
| **All cancers** | 31 | 33 | 93 (66 - 133) | 89 | 105 | 85 (69 - 105) |
| **Digestive Organs** | <6 |  | 73 (24 - 227) | 13 | 12 | 111 (65 - 192) |
| Colorectal | <6 |  | 36 (5 - 252) | 7 | 8 | 85 (41 - 179) |
| Colon | <6 |  | 59 (8 - 416) | <6 |  | 81 (30 - 216) |
| Rectum | 0 |  |  | <6 |  | 71 (18 - 283) |
| **Respiratory and Intrathoracic Organs** | <6 |  | 114 (28 - 455) | <6 |  | 91 (34 - 243) |
| Lung | <6 |  | 121 (30 - 486) | <6 |  | 99 (37 - 263) |
| **Melanoma** | <6 |  | 74 (24 - 230) | 16 | 14 | 115 (70 - 188) |
| **Breast** | 14 | 12 | 118 (70 - 199) | 25 | 38 | 65 (44 - 97) |
| **Female Reproductive Organs** | <6 |  | 82 (26 - 253) | 6 | 12 | 52 (23 - 115) |
| Cervix | <6 |  | 195 (49 - 781) | <6 |  | 25 (4 - 181) |
| **Thyroid and Other Endocrine Glands** | <6 |  | 91 (23 - 364) | 10 | 8 | 126 (68 - 235) |
| Thyroid | <6 |  | 93 (23 - 371) | 10 | 8 | 129 (69 - 239) |
| **Lymphoid, Haematopoietic + Related Tissue** | -<6-- |  | 126 (41 - 392) | 7 | 7 | 95 (45 - 199) |
| Hodgkin | 0 |  |  | <6 |  | 162 (41 - 649) |
| Non-Hodgkin Lymphoma | <6--- |  | 99 (14 - 701) | <6 |  | 66 (16 - 263) |

* Could not be classified to another Work Category e.g. Production or Maintenance

Table S6: Relative Incidence Ratios (RIRs) for overall cancers among women, for each Work Categories compared with the rest of the female cohort not in that Work Category

| Category | Number with cancer (%) | Unadjusted  RIR (95% CI) | Adjusted*  RIR (95% CI) |
| --- | --- | --- | --- |
| ONLY Administration | 142 (2.1) | 1.19 (0.97 - 1.47) | 1.31 (1.05 - 1.63) |
| ONLY Unexposed Non-Office | 28 (1.6) | 1.04 (0.71 - 1.53) | 0.90 (0.61 - 1.33) |
| ONLY Occasionally exposed | 18 (1.2) | 0.76 (0.47 - 1.22) | 1.09 (0.68 - 1.76) |
| EVER Maintenance - all | 20 (1.3) | 0.73 (0.46 - 1.14) | 0.68 (0.42 - 1.09) |
| EVER Production - all | 85 (1.6) | 0.78 (0.61 - 0.99) | 0.86 (0.67 - 1.09) |
| EVER Cleaner** | 60 (2.4) | 1.28 (0.97 - 1.69) | 0.93 (0.70 - 1.24) |

* Adjusted for era of first examination, age and smoking status.

****** Unknown Work Category

Table S7: Relative Incidence Ratios for female Administration and Production workers compared with all other female coal mine workers not in that Work Category (adjusted for age and smoking)

| Cancer Categories | Only Administration vs all other female workers | | Ever Production vs all other female workers | |
| --- | --- | --- | --- | --- |
|  | **Unadjusted**  **RIR (95% CI)** | **Adjusted***  **aRIR (95% CI)** | **Unadjusted**  **RIR (95% CI)** | **Adjusted***  **aRIR (95% CI)** |
| Digestive Organs | 1.18 (0.66 - 2.12) | 1.39 (0.75 - 2.59) | 1.00 (0.53 - 1.88) | 1.13 (0.58 - 2.18) |
| Colorectal | 1.45 (0.73 - 2.90) | 1.75 (0.84 - 3.63) | 0.72 (0.31 - 1.66) | 0.79 (0.34 - 1.85) |
| Respiratory & Intrathoracic Organs | 0.92 (0.38 - 2.26) | 1.28 (0.48 - 3.44) | 0.60 (0.20 - 1.76) | 0.83 (0.28 - 2.44) |
| Lung | 0.99 (0.40 - 2.44) | 1.36 (0.49 - 3.72) | 0.63 (0.21 - 1.88) | 0.88 (0.30 - 2.58) |
| Melanoma | 1.76 (1.10 - 2.81) | 1.70 (1.03 - 2.81) | 0.80 (0.46 - 1.39) | 0.85 (0.48 - 1.51) |
| Breast | 1.30 (0.92 - 1.83) | 1.44 (0.99 - 2.08) | 0.60 (0.39 - 0.93) | 0.70 (0.45 - 1.08) |
| Female Reproductive Organs | 0.77 (0.36 - 1.67) | 0.80 (0.36 - 1.77) | 0.62 (0.25 - 1.50) | 0.68 (0.28 - 1.63) |
| Cervix | 1.32 (0.37 - 4.66) | 1.12 (0.28 - 4.49) | 0.30 (0.04 - 2.35) | 0.34 (0.04 - 2.58) |
| Thyroid and other Endocrine Glands | 0.93 (0.42 - 2.07) | 1.05 (0.47 - 2.34) | 1.49 (0.69 - 3.23) | 1.36 (0.64 - 2.91) |
| Thyroid | 0.88 (0.38 - 2.02) | 0.98 (0.43 - 2.24) | 1.68 (0.76 - 3.70) | 1.53 (0.70 - 3.32) |
| Lymphoid, Haematopoietic + Related Tissue | 1.97 (0.92 - 4.26) | 2.01 (0.91 - 4.41) | 0.99 (0.42 - 2.35) | 1.11 (0.48 - 2.59) |
| Hodgkin | 1.48 (0.33 - 6.61) | 1.42 (0.38 - 5.30) | 1.07 (0.21 - 5.53) | 1.11 (0.23 - 5.30) |
| Non-Hodgkin Lymphoma | 3.45 (1.01 - 11.8) | 3.27 (0.94 - 11.35) | 0.60 (0.13 - 2.76) | 0.77 (0.16 - 3.72) |
| Leukaemia | 0.99 (0.18 - 5.38) | 1.25 (0.20 - 7.67) | 1.34 (0.25 - 7.33) | 1.76 (0.43 - 7.16) |

Table S8: Over view of cancer risk for men in the cohort by Work Category compared to General Population standardised for age and era (no brackets) and within cohort comparisons to workers not in that Work category (in round brackets) after adjusting for age and smoking

| **Type of Cancer** | **Administration** | **Unexposed non-Office** | **Occasionally exposed*** | **Maintenance** | **Production** | **Exploration Driller*** | **Construction** | **Truck Driver** | **Labourer** | **All Men** |
| --- | --- | --- | --- | --- | --- | --- | --- | --- | --- | --- |
| **All Malignancies** | =** ()** | **** | = | =** ()** | ** ()** | = | = | **** | **** | **** |
| **Lip, Oral Cavity etc.** | = | = | = | **** | **** | **** | = | = | = | **** |
| Lip | = | = | = | **** | ** ** | **** | = | **** | = | **** |
| Pharynx | = | = | = | = | = | = | ** ()** | = | = | = |
| **Digestive Organs** | = | ** (η)** | = | = | = | **** | = | = | = | **** |
| Colorectal | = | **** | = | = | ** (η)** | 0 | = | = | = | = |
| Gallbladder | = | = | = | **** | = | 0 | = | = | = | **** |
| **Respiratory** | = | = | = | = | ** (η)** | = | ** ()** | = | = | **** |
| Larynx | = | = | = |  | **** | 0 | = | = | 0 | **** |
| Lung | = | = | = | = | ** (η)** | = | ** ()** | = | = | = |
| **Melanoma** | **** | = | **** | ** ()** | **** | **** | = | **** | = | **** |
| **Mesothelioma** | **** | 0 | **** | **** | = **()** | 0 | = | 0 | = | **** |
| **Male Reproductive** | = | = | = | ** ()** | ** ()** | = | = | **** | = | **** |
| Prostate | = | = | = | **** | ** ()** | = | = | ** (η)** | **** | **** |
| Testis | = | ** ()** | = | **** | = | = | = | = | = | = |
| **Urinary Tract** | = | = | = | = | ** (η)** | = | = | = | = | **** |
| Kidney | = | = | = | = | ** (η)** | = | = | = | = | = |
| **Brain & CNS** | = | = | = | = | = | = | = | = | = | **** |
| Brain | = | = | = | = | **** | = | = | = | = | **** |
| **Thyroid & Endocrine** | = | = | = | = | = | = | = | = | = | = |
| **Lymphoid, Haema-topoietic + Related Tissue** | =** ()** | = | = | = | **** | = | ** ()** | = | = | **** |
| NHL | =** ()** | = | = | = | = | = | **** | = | = | **** |
| Leukaemia | = | = | = | = | = | 0 | = | ** (η)** | = | **** |
| Lymphoid leukaemia | = | = | = | **** | **** | 0 | = | **** | **** | **** |
| Myeloid leukaemia | = | = | = | = | **** | 0 | 0 | = | 0 | = |
| **Other Cancers** | = | = | = | = | ** ()** | = | = | = | = | = |

* No within cohort analyses because of small numbers.

Key

| **** | Statistically significant increase | **** | Non-statistically significant increase *(estimate ≥110 & CI lower limit > 90)* | = | No difference |
| --- | --- | --- | --- | --- | --- |
| **** | Statistically significant decrease | **** | Non-statistically significant decrease *(estimate <95 & CI upper limit ≤ 105)* | 0 | No cases |
